# Assessment of DNA extracted from FTA^® ^cards for use on the Illumina iSelect BeadChip

**DOI:** 10.1186/1756-0500-2-107

**Published:** 2009-06-16

**Authors:** Matthew C McClure, Stephanie D McKay, Robert D Schnabel, Jeremy F Taylor

**Affiliations:** 1 Division of Animal Sciences, University of Missouri-Columbia, Columbia, MO, USA

## Abstract

**Background:**

As FTA^® ^cards provide an ideal medium for the field collection of DNA we sought to assess the quality of genomic DNA extracted from this source for use on the Illumina BovineSNP50 iSelect BeadChip which requires unbound, relatively intact (fragment sizes ≥ 2 kb), and high-quality DNA. Bovine blood and nasal swab samples collected on FTA cards were extracted using the commercially available GenSolve kit with a minor modification. The call rate and concordance of genotypes from each sample were compared to those obtained from whole blood samples extracted by standard PCI extraction.

**Findings:**

An ANOVA analysis indicated no significant difference (P > 0.72) in BovineSNP50 genotype call rate between DNA extracted from FTA cards by the GenSolve kit or extracted from whole blood by PCI. Two sample t-tests demonstrated that the DNA extracted from the FTA cards produced genotype call and concordance rates that were not different to those produced by assaying DNA samples extracted by PCI from whole blood.

**Conclusion:**

We conclude that DNA extracted from FTA cards by the GenSolve kit is of sufficiently high quality to produce results comparable to those obtained from DNA extracted from whole blood when assayed by the Illumina iSelect technology. Additionally, we validate the use of nasal swabs as an alternative to venous blood or buccal samples from animal subjects for reliably producing high quality genotypes on this platform.

## Background

The advent of high-throughput SNP genotyping has revolutionized our ability to obtain high density genotypes, however, a key issue remains; the need to access, store, and extract DNA from each individual. While DNA collected for SNP analysis needs to be of sufficient quality to ensure high genotype call rates, the method of collection used in the field needs to be straightforward. FTA filter paper cards (Whatman, Part of GE Healthcare, Florham Park, NJ, USA) simplify the harvesting and storing of samples, and once properly dried they can be stored at room temperature for years without DNA deterioration [1]. While the chemically infused paper kills microorganisms and prevents degradation of the matrix-bound DNA [2], the bound DNA must be extracted and resuspended in an aqueous solution before it can be genotyped by high-throughput SNP genotyping platforms, such as the Illumina iSelect BeadChip (San Diego, CA, USA).

Previous research has shown that multiple genomic sources, including lymphocytes, buccal cells, whole genome amplified samples, and fingernails can be used to generate high-density SNP data provided the DNA sample is of adequate quality and quantity [3-6]. While venous blood is often considered an optimal source for DNA, the invasiveness and cost of obtaining venous blood samples can be prohibitive [3,7], especially for large-scale studies or those that deal with livestock and wild animals. Additionally, fresh samples collected in the field may experience degradation before they can be processed [2]. The ease of collection, transportation, storage, and protection from degradation of samples stored on FTA cards alleviates many of these issues [8].

While previous studies have shown that DNA harvested from FTA cards is suitable for genotyping 1,516 SNP on the Illumina GoldenGate platform and 10,000 SNP on the Affymetrix 10 K GeneChip Human Mapping 10 K Array XBA 142 2.0 [9,10], it is not known if these samples are appropriate for high-throughput genotyping on the Illumina iSelect platform, which currently assays up to 200,000 SNP [11]. To determine the utility of FTA cards as a collection and storage media for DNA analyzed by iSelect BeadChips which requires unbound, relatively intact (fragment sizes > 2 kb), and high-quality DNA [12], we analyzed the call rate and concordance of 54,122 SNP genotypes produced by the Illumina BovineSNP50 BeadChip [13]. Whole blood and nasal swabs were collected on FTA and FTA Elute cards and DNA was harvested from the cards using a minimally modified GenSolve protocol (GenVault, Carlsbad, CA, USA). Genotypes produced from these samples were benchmarked against genotypes produced from DNA extracted directly from buffy coats by proteinase K treatment, PCI extraction, and ethanol precipitation [14].

## Methods

The following samples were collected from two Angus (*Bos taurus*) bulls: 10 ml of whole blood (WB) collected and stored in vacuum tubes with 15 mg of EDTA (Covidien, Mansfield, MA, USA), WB was also collected from ear veins and applied to FTA and FTA Elute cards [15,16], and nasal swab samples were collected using a sterile foam tipped applicator (Whatman) which was rubbed for 10 seconds against the inside of the bull's nose and then pressed against an FTA Elute card to transfer cells to the card.

Buffy coats were isolated from each of the 10 ml WB samples and DNA was extracted by proteinase K treatment followed by PCI extraction and ethanol precipitation [14]. Genotypes produced from these DNA samples were used as the standards against which genotypes produced from samples harvested from the FTA cards were compared. DNA was extracted from 3 mm punches obtained from each FTA and FTA Elute card using a GenSolve kit (GenVault). We minimally modified the manufacturer's protocol by using a PCI extraction and ethanol precipitation instead of a Qiagen kit for DNA cleanup, [see Additional file 1]. Three hundred nanograms of DNA from each extraction was used as template for the BovineSNP50 BeadChip, which was processed and analyzed according to Illumina's protocol for the iSelect single base extension reaction [12].

A one-way ANOVA was performed on BovineSNP50 BeadChip call rates from FTA extracted samples and those achieved from assaying 7,737 *Bos taurus *samples extracted from WB or cryopreserved semen by PCI extraction in our laboratory (Table 1). Thirty-five of these samples had two aliquots individually genotyped on the BovineSNP50 BeadChip which generated technical replicates that we used to calculate baseline concordance values. Genotypes produced from each FTA extracted DNA sample were compared for concordance to those obtained from WB for each animal. Call and concordance rates were analyzed with a two sample t-test assuming equal within-treatment variances (Table 2).

**Table 1:** One-way ANOVA comparing call rates for BovineSNP50 genotypes produced from DNA extracted by the GenSolve kit from blood and nasal swabs harvested on FTA cards to 7,737 samples extracted from whole blood or cryopreserved semen extracted by proteinase K treatment, Phenol:Chloroform:Isoamyl alcohol extraction, and ethanol precipitation.

SUMMARY						
*Groups*	*Count*	*Sum*	*Average*	*Variance*		

PCI	7737	7648.8155	0.9886	0.0015		
Blood FTA	2	1.9807	0.9904	5.17E-06		
Blood FTA Elute	2	1.9687	0.9844	6.97E-06		
Nasal FTA Elute	2	1.9157	0.9578	0.0020		

						
ANOVA						

*Source of Variation*	*SS*	*df*	*MS*	*F*	*P-value*	*F crit*

Between Groups	0.0019	3	0.0006	0.4414	0.7234	2.6061
Within Groups	11.3121	7739	0.0015			
						
Total	11.3141	7742				

**Table 2:** Genotype call and concordance percentage rates for DNA samples extracted from FTA cards, by sample type.

	Standard	Blood FTA	Blood FTA Elute	Nasal Swab FTA Elute
Call Rate %	98.86	99.04 (0.07)	98.44 (0.44)	95.78 (0.25)

Concordance Rate %	99.006	99.817 (0.40)	99.786 (0.40)	99.573 (0.43)

Alternative Homozygous Rate %	0.006	0.000 (0.40)	0.000 (0.40)	0.002 (0.43)

Homozygous vs. Heterozygous Rate %	0.988	0.183 (0.40)	0.214 (0.40)	0.425 (0.43)

Two sample t-tests assuming equal within-treatment variances, the number in parenthesis is the P-value corresponding to the comparison of that sample to the standard. Two samples from each FTA type were compared to 7,737 samples extracted by PCI for call rate and to 35 samples extracted by PCI for which dual aliquots were genotyped on the Bovine SNP50 BeadChip for concordance, alternative homozygous, and homozygous vs. heterozygous rate.

## Results

The ANOVA indicated no statistical difference in call rate (P > 0.72) due to method of DNA extraction, FTA card type, or sample type (Table 1). We were concerned whether genotypes obtained from DNA harvested from FTA cards would yield reproducible genotypes that were highly concordant with those produced from DNA extracted from WB. Table 2 shows that > 99% of called genotypes were concordant for every sample type and that discordances were primarily between the homozygous vs. heterozygous genotype classes. In every concordance comparison, genotypes from DNA samples harvested from FTA cards were not different from those produced from the standard samples (P > 0.40).

## Conclusion

This report shows that blood and nasal swab samples stored on FTA cards can be processed in a manner that results in high-quality DNA capable of producing robust results on Illumina's iSelect BeadChips. While only the BovineSNP50 BeadChip was tested, similar results should be obtainable on other iSelect BeadChips such as the CanineSNP20, EquineSNP50, OvineSNP50, and PorcineSNP60. As DNA yields from individual FTA card punches vary between samples [8,17], and our FTA samples ranged in yield from 101 to 405 nanograms of DNA per punch, we recommend that at least six 3 mm punches be extracted per sample to ensure sufficient DNA for genotyping. Assuming sufficient quantities are obtained, we speculate that DNA extracted from FTA cards by the GenSolve kit will also produce quality genotypes on other high-density SNP platforms such as Affymetrix Genome-Wide Human SNP Array 6.0 genechip and the Illumina Human1M-Duo BeadChip which both assay over 1 million SNP, although further studies are needed for confirmation due to the different chemistries used on each platform [18,19].

We conclude that FTA cards provide an excellent medium for harvesting DNA from multiple tissue types, and that when assayed using the Illumina iSelect technology, yield high genotype call rates and reproducibility, particularly when the DNA is extracted using the GenSolve kit. By demonstrating that high quality and repeatable genotypes can be obtained from DNA stored on FTA cards, we alert the community to the utility of this sample storage medium for DNA intended for high-throughput SNP genotyping.

## List of Abbreviations

DNA: Deoxyribonucleic acid; FTA: Flinders Technology Associates; PCI: Phenol:Chloroform:Isoamyl alcohol; SNP: single nucleotide polymorphism.

## Competing interests

The authors declare that they have no competing interests.

## Authors' contributions

MCM: Participated in the design of the study, obtained tissue samples and extracted DNA, and drafted the manuscript. SDM: Performed the Illumina BovineSNP50 BeadChip assays and manuscript writing. RDS: Scored BovineSNP50 BeadChip genotypes and manuscript writing. JFT: Assisted with experimental design and manuscript writing. All authors have read and approved the final manuscript.

## Supplementary Material

Additional file 1 **Modified GenSolve DNA Extraction from FTA Cards for Illumina iSelect BeadChip Genotyping**. DNA extraction protocol used for extracting genomic DNA from FTA cards using the GenSolve kit. Click here for file

## References

[B1] Ledray LE, Netzel L (1997). DNA evidence collection. J Emerg Nurs.

[B2] Smith LM, Burgoyne LA (2004). Collecting, archiving and processing DNA from wildlife samples using FTA databasing paper. BMC Ecol.

[B3] Woo JG, Sun G, Haverbusch M, Indugula S, Martin LJ, Broderick JP, Deka R, Woo D (2007). Quality assessment of buccal versus blood genomic DNA using the Affymetrix 500 K GeneChip. BMC Genet.

[B4] Montgomery GW, Campbell MJ, Dickson P, Herbert S, Siemering K, Ewen-White KR, Visscher PM, Martin NG (2005). Estimation of the rate of SNP genotyping errors from DNA extracted from different tissues. Twin Res Hum Genet.

[B5] Feigelson HS, Rodriguez C, Welch R, Hutchinson A, Shao W, Jacobs K, Diver WR, Calle EE, Thun MJ, Hunter DJ (2007). Successful genome-wide scan in paired blood and buccal samples. Cancer Epidemiol Biomarkers Prev.

[B6] Nakashima M, Tsuda M, Kinoshita A, Kishino T, Kondo S, Shimokawa O, Niikawa N, Yoshiura K (2008). Precision of high-throughput single-nucleotide polymorphism genotyping with fingernail DNA: comparison with blood DNA. Clin Chem.

[B7] Saab YB, Kabbara W, Chbib C, Gard PR (2007). Buccal cell DNA extraction: yield, purity, and cost: a comparison of two methods. Genet Test.

[B8] Vidal-Taboada JM, Cucala M, Mas Herrero S, Lafuente A, Cobos A (2006). Satisfaction survey with DNA cards method to collect genetic samples for pharmacogenetics studies. BMC Med Genet.

[B9] Whatman Inc FTA Elute Data Sheet. http://www.whatman.com/References/FTA%20Elute%20Data%20Sheet(1).pdf.

[B10] Whatman Inc DNA Extraction from FTA^® ^Cards Using the GenSolve DNA Recovery Kit. http://www.whatman.com/References/GenSolveDataSellSheetLR.pdf.

[B11] Illumina Inc News Release: Illumina Announces High Density Expansion to iSelect(R) Custom Genotyping Platform. http://investor.illumina.com/phoenix.zhtml?c=121127&p=irol-newsArticle&ID=1243814&highlight=.

[B12] Steemers FJ, Chang W, Lee G, Barker DL, Shen R, Gunderson KL (2006). Whole-genome genotyping with the single-base extension assay. Nat Methods.

[B13] Matukumalli LK, Lawley CT, Schnabel RD, Taylor JF, Allan MF, Heaton MP, O'Connell J, Moore SS, Smith TPL, Sonstegard TS, Van Tassell CP (2009). Development and characterization of a high density SNP genotyping assay for cattle. PLoS One.

[B14] Sambrook J, Fritsch EF, Maniatis T (1989). Molecular Cloning: A Laboratory Manual.

[B15] McClure MC, Weaber R, Olson KC (2005). Collecting Genetic Material from Beef Cattle, Guidesheet G2140.

[B16] Whatman Inc FTA for Collecting Cattle DNA. http://www.whatman.com/References/51668.pdf.

[B17] Harty LC, Garcia-Closas M, Rothman N, Reid YA, Tucker MA, Hartge P (2000). Collection of buccal cell DNA using treated cards. Cancer Epidemiol Biomarkers Prev.

[B18] Affymetrix Inc Data Sheet: Affymetrix^® ^Genome-Wide Human SNP Array 6.0. http://www.affymetrix.com/products_services/arrays/specific/genome_wide_snp6/genome_wide_snp_6.affx.

[B19] Illumina Inc Genome-Wide DNA Analysis BeadChips. http://www.illumina.com/downloads/InfiniumHD_DataSheet.pdf.

