# Supplementary material for: Assessment of DNA extracted from FTA® cards for use on the Illumina iSelect BeadChip
Source: BMC Res Notes. 2009 Jun 16;2:107. doi: 10.1186/1756-0500-2-107 (PMC2704227; doi:10.1186/1756-0500-2-107)
Supplement: Additional file 1 — Modified GenSolve DNA Extraction from FTA Cards for Illumina iSelect BeadChip Genotyping. DNA extraction protocol used for extracting genomic DNA from FTA cards using the GenSolve kit. [file 1756-0500-2-107-S1.doc]

Supplemental Material

**Modified GenSolve DNA Extraction from FTA Cards for Illumina iSelect BeadChip Genotyping.**

*Matthew McClure, Stephanie McKay, Robert Schnabel, Jeremy Taylor*

*Division of Animal Sciences, University of Missouri, Columbia, MO, USA 65211*

**REAGENTS**

Chloroform (Thermo Fisher Scientific Inc, Waltham, MA, USA)

Ethanol, absolute (Thermo Fisher Scientific Inc)

Phenol:Chloroform:Isoamyl Alcohol (24:24:21) (Thermo Fisher Scientific Inc)

Ammonium Acetate (Thermo Fisher Scientific Inc)

Tris (Thermo Fisher Scientific Inc)

EDTA (Thermo Fisher Scientific Inc)

GlycoBlue (Ambion, Austin, TX, USA)

Recovery Solution A (GeneVault, San Diego, CA, USA)

1.0% LiDS Solution (GeneVault)

Recovery Solution B (GeneVault)

**PROCEDURE**

Obtain punches:

1. Punch six 3 mm circles from an FTA or FTA Elute card and place into a sterile 2.0 ml tube.

2. Punch a clean sheet of filter paper 3× to prevent cross contamination between samples.

Begin extraction: Adopted from GenVault protocol for the GenSolve kit.

3. Reconstitute Recovery Solution A with 6 ml of 1.0 % LiDS solution and vortex to resuspend.

4. Add 200 µl of Protease and vortex briefly.

5. Aliquot 620 µl of the Recovery Solution A/Protease mixture into each tube.

6. Centrifuge briefly to completely submerge the punches.

7. Incubate in a thermomixer at 65 °C for 1 hour at a constant vortex of 1,400 rpm.

8. Centrifuge at 16,000×g for 30 seconds to collect condensation.

9. Place 20 µl of Recovery Solution B and a DNA IQ Spin Basket into new 2.0 ml tubes.

10. Transfer the solution and punches from the initial tube and place into the spin basket.

12. Centrifuge at 16,300×g for 2 minutes.

13. Discard the spin baskets and punches.

Phenol-Chloroform Extraction:

14. Extract with 25:24:21 PCI and with CHCl_3_ as follows:

a. Add 700 μl of PCI and mix by inversion for 5 minutes.

b. Spin for 5 minutes at 6,000×g.

c. Transfer the top aqueous layer to new labeled tubes.

d. Add 600 μl of CHCl_3_ and mix by inversion for 5 minutes.

e. Spin for 5 minutes at 6,000×g.

f. Transfer the top aqueous layer to new labeled tubes.

g. Repeat steps d-f once if interface is not clean.

Ethanol Precipitation:

15. Add 0.2 volumes of 10 M ammonium acetate and 0.50 μl of GlycoBlue.

16. Add 2 volumes of cold absolute ethanol.

17. Mix by inversion.

18. Store tubes at -20 °C for 2 to 16 hours.

19. Spin for 5 minutes at 16,000×g.

20. Pour off ethanol, taking care to leave the DNA pellet.

21. Add 700 μl of 70 % ETOH.

22. Mix by inverting the tubes.

23. Spin for 5 minutes at 16,000×g.

24. Pour off 70 % ethanol taking care to leave the DNA pellet.

25. Air dry DNA pellet completely.

26. Reconstitute with 50 µl of Tris-EDTA or ddH_2_O.

**RECIPES**

Ammonium acetate (100 ml)

Ammonium acetate 77.08 g 10 M

ddH2O 30.00 ml

Place ammonium acetate into a beaker containing a stir bar.

Add the water and stir.

Once all of the ammonium acetate is in solution bring the total volume up to 100 ml with ddH_2_O.

70 % ETOH (50 ml)

100 % ETOH 35 ml 70 % (v/v)

ddH_2_0 15 ml

Combine in a 50 ml conical tube and invert to mix.

Tris-EDTA (1 L)

EDTA 0.372 g

Tris 12.114 g

Combine EDTA and Tris in a beaker containing a stir bar.

Add 600 ml of ddH_2_O and stir.

After EDTA and Tris are in solution bring the total volume to 1 L with ddH_2_O.

pH to 8.0.

**EQUIPMENT**

Thermomixer (Eppendorf, Hamburg, Germany)

SmartSpec 3000 (BioRad, Hercules, CA, USA)

Centrifuge 5415D (Eppendorf)

DNA IQ Spin Basket (Promega, Madison, WI, USA)

2.0 ml centrifuge tubes (Thermo Fisher Scientific Inc)
